# Understanding sex and gender disparities in COVID-19 mortality: a narrative review beyond biology

**DOI:** 10.1186/s13293-025-00762-z

**Published:** 2025-10-15

**Authors:** Patricia Lemarchand, Madeleine Pape, Joëlle Schwarz

**Affiliations:** 1 Nantes Université, CHU Nantes, CNRS, INSERM, l’institut du thorax, Nantes, F-44000 France; 2 Institute of Social Sciences, University of Lausanne, Géopolis, Quartier Mouline, 1015 Lausanne Switzerland; 3 Healthand Gender Unit, Unisanté, University Center for Primary Care and Public Health & University of Lausanne, Lausanne, Switzerland; 4 l’institut du thorax, UMR Inserm 1087, CNRS 6291, IRS-UN, 8 quai Moncousu, BP 70721, Nantes cedex 1, 44007 France

**Keywords:** COVID-19, Mortality, Sex, Gender, Social determinants of health, Comorbidities, Structural inequalities, Public health, Entanglement

## Abstract

**Background:**

Men have consistently experienced higher COVID-19 mortality than women across most countries and time periods, prompting widespread investigation into potential biological causes. Early research focused on sex-related genetic, hormonal, and immunological mechanisms to explain these disparities.

**Main body:**

This narrative review traces the evolution of scientific explanations for women/men mortality differences in COVID-19, from early biological hypotheses to more nuanced gendered and intersectional models. While some studies suggest sex-linked genetic variants, chromosomal mechanisms, or hormone-regulated expression of viral entry receptors might partially explain men’s higher mortality, the overall evidence remains inconsistent and inconclusive. Increasingly, attention has shifted to social and structural factors, including occupational exposure, pre-existing health conditions, healthcare access, and behaviors, that can differently shape vulnerability to COVID-19 in women and men. Data disaggregated by gender and race further revealed significant heterogeneity in outcomes, underscoring the influence of intersecting axes of inequality. International comparisons suggested that gender inequality at the societal level was associated with wider women/men COVID-19 mortality gaps. Analyses that overlook the interaction between sex- and gender-related factors and their intersection with racial disparities and socioeconomic status risk obscuring the underlying drivers of COVID-19 disparities.

**Conclusion:**

Sex-related biological factors may have influenced COVID-19 outcomes, but they do not adequately account for the observed differences in mortality between women and men. Approaching the study of health inequities through a gendered, intersectional framework is essential for accurately identifying and addressing underlying risk factors, and for better understanding how sex- and gender-related factors may interact, not only in COVID-19, but across a broad range of health conditions.

## Highlights

- Men have experienced higher COVID-19 mortality than women across most settings, but the magnitude and consistency of this disparity vary widely by age, time, location, and population group.
- Biological explanations, including genetic, hormonal, and immunological factors, have yielded mixed, inconsistent, and often inconclusive results.
- Gendered behaviors, occupational roles, comorbidities, and structural inequalities play a key role in shaping COVID-19 outcomes.
- Intersectional analyses reveal that social determinants, particularly socioeconomic status and race, modify disparities in COVID-19 mortality between women and men.
- These findings support a broader application of gendered and intersectional approaches across biomedical research and public health, as well as approaches that consider the interaction of sex- and gendered-related factors.

## Background

From the earliest months of the COVID-19 pandemic, a striking and consistent pattern emerged worldwide: men were more likely than women to die from SARS-CoV-2 infection. This men’s higher COVID-19 mortality, observed across continents and age groups, initially suggested a biological explanation rooted in genetic, hormonal, or immunological sex differences. Consequently, early research focused predominantly on biological sex as a determinant of COVID-19 outcomes, generating a large body of work exploring chromosomal, hormonal, and immune mechanisms. However, as the pandemic progressed, it became increasingly clear that social, economic, and gendered factors also played a crucial role in shaping individual risk. Health behaviors, occupational exposure, pre-existing health disparities, and structural inequalities intersected with sex and gender in complex ways that the binary categories of male and female could not fully explain.

In this narrative review, we explore the multiple and often overlapping hypotheses, although mostly divided as “biological” and “non-biological”, that have been proposed to account for men’s higher COVID-19 mortality. Our aim is not to provide an exhaustive list of publications, but rather to offer a structured synthesis that reflects the chronological evolution of the scientific discourse, from early biological models on the one side, toward models that incorporate gendered and intersectional inequalities on the other. We note that neither, however, appears to tell the full story of COVID-19 mortality disparities, pointing to an opportunity to approach sex/gender from a perspective that assumes their entanglement [1]. At the same time, we observe that whereas research focusing on the biological sex hypothesis has attracted considerable investment and attention, there are many remaining avenues to be explored before it could be said that the social environment hypothesis has been exhausted.

Literature from this review is assumed to mostly use a conceptualization of sex referring to biological characteristics (including genetic, hormonal, physiological, anatomical) that distinguish between male, female, and intersex (in humans), and gender referring to socio-cultural norms, identities and relations that, together, shape and sanction what are considered “feminine” and “masculine” behaviors, and structure societies and organizations [2]. In the COVID-19 literature, sex/gender is predominantly reported and analyzed as a binary categorical variable, with limited or no information regarding how sex- and/or gender-related data were collected. As a result, these existing studies may very well include individuals with variations in sex characteristics, transgender people, or people with diverse gender identities. At the very least, the studies reviewed are relevant to such groups, though further research is needed to clarify specific health outcomes and risks for these individuals.

## The initial findings: higher mortality among men

By April 2020 of the COVID-19 pandemic, demographic data on cases and deaths linked to SARS-CoV-2 infection revealed a striking trend: everywhere, men were dying in greater numbers than women [3]. While women accounted for the majority of confirmed cases, follow-up registries confirmed that men represented the majority of deaths, resulting in a higher case-fatality rate among men - with odds ratios as high as 2.15 reported in August 2020 [4] - and suggesting that they developed more severe forms of the disease [5]. A similar pattern was observed in a large U.S. cohort of hospitalized COVID-19 patients: men experienced more complications, required more frequent ICU admission and mechanical ventilation, and had higher mortality than women, regardless of age [6]. Although in some cohorts, while the rate of death in ICU was higher in men, the overall death rate did not differ between men and women [7].

These early findings were quickly supported by a meta-analysis of over 3 million patients from 46 countries, which showed that men were significantly more likely than women to be admitted to intensive care units and to die following SARS-CoV-2 infection (odds ratio = 1.39) [8]. These observations led to a strong call for action for sex-disaggregated data reporting [3, 9–11]. Global Health 50/50, an independent initiative based at University College London, played a key role through its Sex-Disaggregated COVID-19 Data Tracker [12]. Drawing from governmental surveillance systems in over 170 countries in 2021, the tracker highlighted significant women/men (W/M^1^) disparities in COVID-19 outcomes, showing that men faced consistently higher risks of severe illness and mortality, with death rates over one-third higher than those of women in 79 countries [13]. Updated results in 2022 confirmed these trends: across 28 countries, men were more likely to be hospitalized (men/women (M/W) ratio = 1.21/1), twice as likely to be admitted to intensive care (M/W = 1.91/1; *n* = 19 countries), and more likely to die (M/W = 1.28/1; *n* = 107 countries) [14].

This excess mortality was particularly observed in men with pre-existing co-morbidities and those in older age groups [15], though with notable exceptions: one study in the U.S., for example, reported a larger W/M gap in mortality among adults under < 65 years than among older adults (≥ 65 years) [16]. Another study of 7 countries showed that the difference in case fatality rates ranged from 1.5 in men aged 80+, to over 2 in the 40–49 and 50–59 age groups [17].

**Fig. 1:**
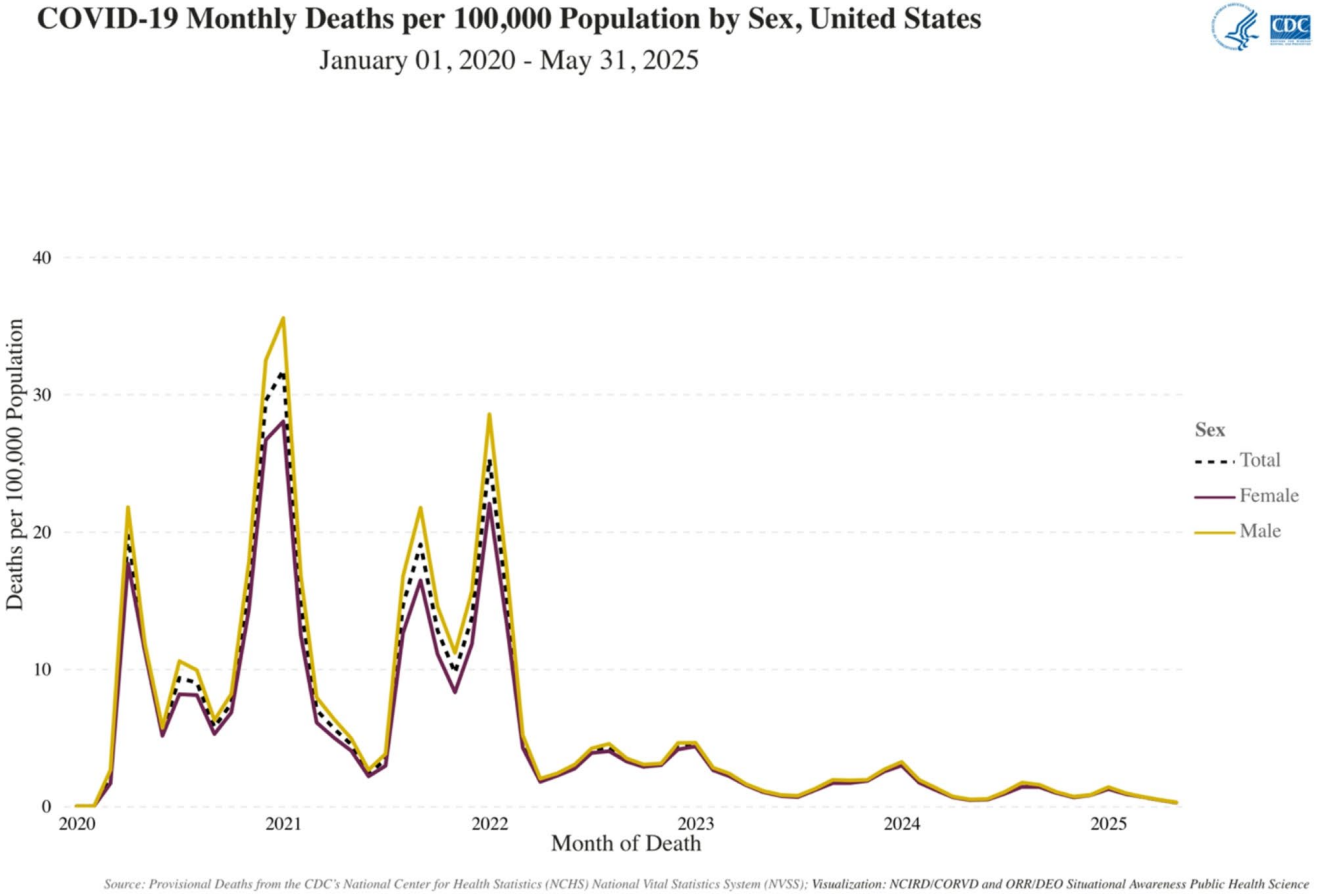
CDC tracker showing COVID-19 monthly deaths per 100 000 population in the U.S., from 01/31/2020 to 05/31/2025 [19]. The mortality rate is shown as a solid brown line for women, a solid yellow line for men, and a black dashed line for the total population

These differences in W/M mortality have been confirmed over the whole epidemic duration, as shown in a recent study on Mexico City population [18], or in the U.S. data from the CDC [19] (Fig. 1).

Nevertheless, the first longitudinal analysis of W/M disparities in COVID-19 cases and deaths across U.S. states, using 13 months of data from the U.S. Gender/Sex COVID-19 Data Tracker, showed extensive heterogeneity in the magnitude and direction of W/M disparities in cumulative mortality, both across states and over time [20]. For instance, men mortality rates were consistently higher in Texas, fluctuated in Connecticut, and in New York dropped from a 1.56 M/W ratio during the first wave to 1.1 by May 2020.

Variation was similarly observed in Europe, where although there was higher COVID-19 mortality in men, peaking in April 2020 before declining and stabilizing by June [21, 22], the comparative men/women mortality rate varied by country, ranging from 1.4 to 1.8, highest in France and England and Wales, and lowest in Belgium and Scotland [21]. Urbanization also showed a significant correlation with COVID-19 W/M mortality ratio, with higher levels of urbanization associated with lower mortality disparities between women and men [14]. A recent international, multicenter, retrospective study of over 10,000 patients hospitalized in the ICU for COVID-19, after adjustment for age, obesity, use of extracorporeal circulation and country of hospitalization, found only a 16% difference in W/M mortality, significantly lower than the above figures [23].

In summary, although men consistently experienced higher COVID-19 mortality than women, this W/M disparity varied notably across time and regions and appears to have been mediated by multiple factors [24]. While early data showed striking differences, with men as much as twice as likely to die, later analyses revealed that the magnitude of this gap fluctuated depending on age, location, healthcare access, and timing within the pandemic. Taken together, these variations point to a role for broader demographic, social, systemic, and other contextual factors in shaping the distribution of COVID-19 outcomes between women and men as well as within these categories.

## The biological sex hypothesis

When average differences are observed between women and men in healthcare settings, researchers and practitioners trained in the biological sciences can be quick to conclude that such differences reflect fundamental, biological differences between female and male bodies [25, 26]. This has been true in the case of COVID, where claims of biological sex differences in genetics and hormones have emerged as a preferred, if contested, explanation for the apparently worldwide higher rate of mortality in men [27–30]. Despite animal models often being poor approximations of human health [31], this biological sex hypothesis has leaned on research showing that male mice and hamsters are more susceptible to SARS-CoV and SARS-CoV-2 infection and related pathology as compared to female animals [32–34].

Despite the complex patterns in COVID-19 mortality, which are far from suggesting that women and men have distinct experiences of the infection, the biological sex hypothesis has led some researchers to claim that women and men may need “sex-specific” treatment and vaccination strategies. In August 2020, for example, Takahashi et al. published a study in *Nature* on the immune phenotype of a small cohort of COVID-19 patients, concluding that “the immune landscape in patients with COVID-19 is considerably different between the sexes” and that “a sex-based approach to the treatment and care of male and female patients” may be justified [35]. This claim was quickly amplified in major media outlets (e.g., *The New York Times*) and echoed in the scientific literature [36, 37], notably in reviews compiling biological hypotheses to explain W/M mortality differences [5]. More than a year later, however, *Nature* published a counter-analysis of this first study [38], which showed that Takahashi et al.’s conclusions were not fully supported by their data, overlooked alternative explanations for COVID-19 mortality disparities, and overstated sex-related immune differences. The authors argued that the study should have been treated as exploratory, noting that its results suggested more similarities than differences in immune responses between men and women, and did not provide a sufficient basis to guide clinical practices or public health strategies aimed at reducing gender disparities for COVID-19.

Such critiques have not deterred efforts to identify essential, immutable biological differences between women and men to explain overall observed COVID-19 disparities, particularly in mortality [37, 39]. An array of research has pursued investigating underlying genetic, chromosomal, and hormonal factors, with immunological differences at each stage of SARS-CoV-2 infection to identify W/M differences that were interpreted as downstream effects [5] and amounting to what was sometimes described as “sexual dimorphism” in the COVID-19 immune response [22, 40]. The term “sexual dimorphism” is scientifically inappropriate in this context, as it traditionally refers to distinct, non-overlapping traits between males and females. Its use reinforces a binary view, whereas “sex differences” more accurately capture the continuous and overlapping nature of traits, such as virus cell receptor expression or T- and B-cell function, observed in both women and men [41]. Results for sex-related factors influencing COVID-19 mortality are summarized in Table 1.

### Genetic factors

Early hypotheses suggested that genetic factors could shape the on-average greater severity of COVID-19 outcomes in men, given that the X chromosome harbors a high density of immune-related genes [42, 43]. Individuals with two X chromosomes are functional mosaics for X-linked genes due to X chromosome inactivation [44, 45]. This, combined with other X chromosome inactivation mechanisms such as genes that escape silencing and skewed inactivation, has been suggested to contribute to an immunological advantage in many infections for individuals with XX chromosomes [46].

Among immune-related genes on the X chromosome are those encoding the toll-like receptors TLR7 and TLR8 [47]. These receptors, upon recognizing SARS-CoV-2 ligands, activate transcription factors that trigger proinflammatory mediators involved in the “cytokine storm” during COVID-19 [47]. Loss-of-function variants in the X-linked TLR7 gene have been identified in at least 1% of men with severe COVID-19 under the age of 60, with high penetrance [48]. Although TLR7 appears to have a protective function against COVID-19, association signals for TLR7 were also detected in women-only subgroups, suggesting the need for further research into additional mechanisms beyond X-linked recessive inheritance in people with XY chromosomes [47, 49]. The androgen receptor, a hormone receptor that inhibits antibody production, is also coded on the X chromosome, showing that even the effect of sex hormones can be amplified by the X-linked sex hormone receptor genes [50]. These genetic differences are thought to act either directly, by influencing immune responses and hormone regulation, or indirectly, via specific gene variations [51].

Although early studies showed no W/M differences in genetic factors for susceptibility to COVID-19 [52], the hypothesis of genetic factors remained. For instance, severe COVID-19 cases were reported in men with androgenic alopecia, a condition associated with elevated testosterone and linked to variations in CAG repeats (e.g., short cytosine-adenine-guanine DNA sequences) on the androgen receptor gene [53]. These findings led to speculation that such genetic variations might help explain worse outcomes in some men. Yet, further genetic investigations failed to confirm any robust genetic differences in susceptibility to COVID-19 between women and men [54]. More recent analyses have highlighted complex associations with specific genetic risk factors, without however consistent evidence of a relationship to W/M disparity [55–57].

A more recent development in this field involved mosaic chromosomal alterations, particularly the loss of the Y chromosome (LOY), which has been associated with a 54% increase in COVID-19 mortality [58]. This condition, which is more prevalent in older men, is associated with immune dysfunction and has been tied to increased risk of SARS-CoV-2 infection [58]. It appears frequently in low-density neutrophils in severely ill men with COVID-19, with these LOY-affected immune cells showing dynamic changes during illness and recovery [59]. It is also linked to reduced expression of key interferon-response genes, offering a potential biological mechanism for increased vulnerability to severe disease [58]. However, the specific role of LOY in different immune cells and other infections remains unclear and requires further study [60]. In summary, genetic factors provided limited support for the biological sex hypothesis, offering at best partial insights into why certain people may be at risk of COVID-19 mortality.

### Hormonal factors

Sex hormones are associated with different clinical and immunological outcomes of viral infections, by generally modulating transcriptional and epigenetic processes in innate and adaptive immune cells [61].

Estrogen is considered an immune activator whereas testosterone is generally viewed as an immune suppressor [46]. Since women have on-average higher levels of estrogen than men, it could be hypothesized that hormones offer women greater resilience to COVID-19. Estrogen may offer women an additional advantage in COVID-19 by reducing airway flooding through its regulation of ion channels and airway surface liquid dynamics [62]. However, estradiol levels are highly variable, often overlapping with those of cisgender men early in the menstrual cycle or in post-menopause [63]; they can also vary dynamically for a range of social, behavioral, and medical reasons, including due to hormone therapy [64].

As in the case of genetic research, animal models have been used to investigate the potential effects of hormones in SARS-CoV infection [65]. Male mice were found to be more susceptible to SARS-CoV mortality than females, with greater viral loads and lung inflammation [32]. Interestingly, whereas castration in males had no effect on disease severity, ovariectomy or estrogen receptor blockade in females increased mortality, suggesting a protective role for estrogens in female mice [32].

In the case of humans, two specific mechanisms have been hypothesized to explain the different susceptibility of women and men to COVID-19 mortality: hormonal regulation of the virus cell receptor (Angiotensin-Converting Enzyme 2 (ACE2)), and of its co-receptor (Transmembrane Protease Serine 2 (TMPRSS2)), which allow viruses to enter the cells of infected individuals [51]. For ACE2, expression may be upregulated in some women, either by estrogens or due to escape from X chromosome inactivation, as the ACE2 gene is located on the X chromosome and can be expressed on both alleles in women [44].

The hypothesis of sex hormones as the basis of W/M differences in COVID-19 mortality is complicated by research suggesting that age increases risk in both women and men. Two studies found that pre-menopausal women had lower hospitalization rates, reduced need for respiratory support, and shorter hospital stays compared to post-menopausal women [66, 67]. Conversely, in men, declining testosterone levels with age associated with increased risk of severe COVID-19, along with hyperinflammation [61, 68].

Taken together, these findings have spurred intense investigation into the role of sex hormones, ranging from cellular and animal models to retrospective and prospective human studies [69], leading to the launch of over a dozen randomized clinical trials as early as 2020 and 2021, as well as studies of COVID-19 treatments testing estradiol, progesterone, or anti-androgens [53]. To date, however, many of these trials have been halted or have yielded mixed results [69]. Overall, the sex hormone hypothesis has largely been abandoned, with no clear justification for hormone therapy in the treatment of COVID-19 [69].

### Genetic + hormonal factors

Chromosomes are argued to combine with hormones as sex-related factors contributing to W/M differences in COVID-19 severity and mortality [69]. The ACE2 gene, located on Xp22.2, is an X escape gene (∼15% of X-linked genes escape inactivation), resulting in higher expression in women [44]. This could, in theory, provide women with a double dose of ACE2 to counteract SARS-CoV-2-mediated loss of membrane ACE2 following infection [70]. ACE2 expression is also influenced by estrogen receptor signaling, and SARS-CoV-2 spike protein can bind and modulate these receptors [71]. However, this potential increase in ACE2 expression in women, which would theoretically exacerbate disease severity, is not consistently observed [72]. Estrogens appear to downregulate ACE2 expression, as shown in human bronchial epithelial cell models [73]. ACE2 is also expressed in multiple organs including heart, kidneys, lungs, and testes, adding further complexity [51]. Interactions among these genetic, hormonal, and tissue-specific factors may vary across the lifespan, influenced by hormonal fluctuations [43]. This complexity has led to speculative hypotheses, such as the idea that ACE2 saturation may occur more slowly in women due to a larger “reservoir”, potentially offering protection against multi-organ inflammation [51]. However, this hypothesis is not supported by evidence, with clinical studies indicating no significant differences in pulmonary ACE2 expression among women and men. Comparable infection rates further suggest similar W/M expression of ACE2 [74]. Notably, many studies focus solely on ACE2 expression, which may not reflect protein function, as ACE2 activity is tightly regulated by proteolytic cleavage and microRNAs [70].

Like ACE2, TMPRSS2 is modulated by sex hormones, with research in this case suggesting that lower androgen levels in women potentially limit its expression. One study of Wettstein et al. has suggested that this might serve as another protective factor against COVID-19 [75]. However, while TMPRSS2 plasma levels were higher in men [76], direct comparison of TMPRSS2 protein levels in women’s and men’s lung tissue revealed no difference in expression in healthy individuals [72], with clinical trials with TMPRSS2 inhibitors in women and/or men yielding no significant results [75].

Despite intense research efforts, there is currently no consensus on whether and to what extent potential differences in ACE2 and TMPRSS2 expression contribute to the higher average disease burden of COVID-19 in men compared to women [69]. Contrary to early assumptions that men with COVID-19 have reduced ACE2 activity and a harmful shift in the renin-angiotensin system balance downstream to ACE2 activation, more robust studies showed no major changes in this balance [74]. Clinical trials targeting different parts of the renin-angiotensin system pathway have not shown that this system plays a key role in the acute phase of COVID-19 [74]. There is currently no clear evidence that a sex-specific expression of virus entry receptors accounts for some of the W/M differences observed in COVID-19 [69].

### Immunological factors

It is generally considered that women have a major immunological advantage over men, with sex-related factors suggested to contribute to women’s lower on average infectious disease susceptibility [46]. Many women exhibit stronger innate and adaptive immune responses than men, with higher activity of macrophages, dendritic cells, T cells, and B cells, as well as greater production of type I interferons [77]. This may contribute to stronger immune responses in women, enhancing vaccine responses and pathogen clearance but increasing the risk of autoimmune conditions [45]. Somewhat paradoxically, while women typically mounted stronger antiviral responses, men often exhibited more severe inflammatory responses to COVID-19 [78]; this may be, in part, because testosterone has been shown to upregulate TLR4 signaling in men [47]. A related hypothesis is that this hyperinflammation may reflect a failure in early antiviral immunity, more common in men [53]. Yet, another contradiction emerges: women with severe COVID-19 have demonstrated robust peripheral T-cell responses, which challenges the assumption of weaker immunity in women during severe COVID disease [43].

The full course of SARS-CoV-2 infection has been examined for W/M differences, from viral entry to type I interferon production, pro-inflammatory cytokine production, T-cell activation, monocytes and dendritic cells, lung-resident immune cells, and neutralizing antibody responses [43, 47, 61, 79–82], including deep immunophenotyping using integrated longitudinal multiomics [83] or metabolomic profiles [84]. These studies have identified inconsistent and sometimes contradictory W/M differences, without clear mechanistic links to mortality disparities [43]. As such, these remain speculative [69].

In summary, despite the many efforts to find support for the biological sex hypothesis to explain why men died more often than women from COVID-19, no definitive proof has emerged. While these studies have suggested potential mechanisms, such as differences in immune responses, sex hormone levels, or chromosomal alterations like LOY, their findings remain inconclusive, often contradictory, and sometimes based on exploratory or limited data. While sex-related factors may shape COVID mortality, there is no evidence that essential, biological factors determine the course of the virus or that experiences of the virus are sex-specific. Overall, the biological sex hypothesis is yet to generate a robust, consistent explanation for observed W/M differences in COVID-19 mortality.

## From biological sex to gender

The search for essential biological differences between women and men as the explanation for average differences in illness and disease outcomes has long been critiqued by feminist scholars, both within and outside of the biomedical sciences. Their work has shown that claims of essential W/M differences are very often unsupported by the underlying data: not only is variation more complex than a simple binary [85], but social and contextual factors are always entangled with biology [86, 87]. A recent perspective on enhancing rigor and precision in the study of sex-related variables, highlighted that dividing a sample into W/M groups can introduce perceptual bias, leading to an exaggerated perception of differences between groups and of similarities within groups, while also encouraging the ignorance of potential confounding factors [41].

Similarly, from the beginning of the pandemic, feminist scholars criticized the ignorance of existing evidence on W/M health differences [88] documented in previous pandemics like the 1918 flu [89] and earlier coronavirus outbreaks [90], in which disparities in outcomes were shaped more by comorbidities, gendered occupational exposures, and gendered behaviors than by sex-related biological factors. In the case of the most recent pandemic, although men had higher COVID-19 mortality, this varied by time, place, and racial group, undermining a strictly biological explanation [91]. Broader gendered factors, such as men’s higher baseline mortality, unequal burden of chronic disease, occupational roles, and health-related behaviors, that proved to be important factors [39], were explored at a later stage of the pandemic.

For example, some studies suggest that the disproportionate mortality rate in men could be explained at least in part by their higher frequency of pre-existing diseases considered to be risk factors for severe form of COVID-19 [92] (e.g., cardiovascular disease [40], hypertension, diabetes, metabolic disorders [93], severe liver disease, renal disease, metastatic solid tumor [94], and chronic lung disease [95]). For obesity, the relative effects of a higher BMI on COVID-19 mortality may have been stronger in women than men [96], although this data did not appear to be consistent [97]. Although biological sex is considered to contribute to W/M differences in these comorbidities, gendered behavioral factors also need to be considered and investigated [98]. Smoking was associated with reduced survival in men hospitalized with COVID-19 [99], likely reflecting its role as a marker of underlying comorbidities that contribute to greater disease severity and mortality [100]. A U.S.-based study found that men hospitalized with COVID-19 were more likely to smoke and consume alcohol and drugs compared to women [93]. Furthermore, returning to the discussion of genetics, smoking may increase ACE2 expression, pointing to a possible gene (sex)/behavioral (gender) interaction [99]. Importantly, associations between comorbidities and COVID-19 mortality were more pronounced in women compared to men in some studies, highlighting the multifactorial and context-dependent nature of W/M differences in COVID-19 mortality [94]. In other words, higher rates of COVID-19 mortality in men may be explained by their on-average poorer baseline health, in part due to higher engagement in risk-taking behaviors and lower adherence to protective health-related behaviors.

Men’s higher propensity to engage in risky behaviors was also observed during the pandemic in their lower uptake of protective measures such as physical distancing and mask-wearing. For example, studies in the U.S. and Hong Kong found that men were less likely during the COVID-19 pandemic to wear a mask than women [101], mirroring patterns during previous pandemics [102]. Other behavioral and social patterns may favor women, with previous studies suggesting that women are more likely than men to adhere to preventive health-related behaviors such as hand hygiene practices and seeking preventive care [101, 103]. However, there were also important differences amongst men during the COVID-19 pandemic: one study found that White men in the U.S. were the least likely amongst race/gender groups to wear a mask during the COVID-19 pandemic, followed by White women. Black, Latino, and Asian men were all more likely than both White women and White men to wear a mask [104].

Occupation is another factor that can shape inequality in COVID-19 incidence [6, 105], which itself is strongly correlated with gender, through the mechanisms of the gendered division of labor. The first studies in New York showed that lockdowns increased inequalities because people working in front-line jobs, such as essential retail, delivery, and health-care workers were unable to work from home. People at high risk of exposure were those in close contact with patients or with the public [106]. Here, occupational status became a risk factor for women, given their over-representation in healthcare and essential retail workers. In Spain, women accounted for 73% of infected healthcare workers; in India, they accounted for 38% of cases despite representing less than a third of the workforce; in Wuhan, women nurses faced 2.7 times higher infection risk than doctors, who are mostly men [107]. Aggregated W/M comparisons fail to capture such variation in exposure and risk obscuring sources of structural vulnerability amongst women, and amongst men [108]. Another mechanism related to gendered division of labor was described in Spain and Switzerland, analyzing how different public health responses such as schools and daycare closures led to excess infection cases in working-age and child-bearing age women [109].

## The social environment hypothesis: intersectional positions

Social epidemiology scholars have studied how the social environment is reflected in the distribution of COVID-19 mortality, exploring patterns across race, class and age as a social determinant. As described elsewhere [110], the exploration of social factors is context-specific: U.S. scholars have focused on racial inequalities while European research has rather focused on socioeconomic conditions. Research has indeed shown that socio-economic factors were strongly associated with COVID-19 incidence and mortality, together with race and ethnicity-related disparities.

In Switzerland, it was shown that W/M mortality differences were most pronounced in the most socioeconomically deprived areas, where women had a 68% lower risk of death compared to men [108], while this gap narrowed in more affluent areas, indicating that socioeconomic conditions may be more consequential to men’s vulnerability to severe COVID-19 outcomes than to women’s.

In France, a nationwide study confirmed the role of socioeconomic factors. Using over 71 million test results from May 2020 to April 2021, they found that COVID-19 incidence, positivity, and testing rates varied by social deprivation index (a composite measure of area-level socioeconomic deprivation, combining ten census-based variables that reflect individual deprivation, such as rates of unemployment, low education, non-home ownership, single-parent families, and overcrowded housing), and by geography. People in the most deprived areas were more likely to contract the virus but less likely to be tested, with the social gradient being steeper in densely populated regions [111].

The role of socioeconomic conditions in men’s higher COVID-19 mortality has also been observed on a macro scale: the M/W ratio of deaths was 2.08/1 in low-income countries, falling to 1.21/1 in high-income countries [14], with the exception of India as compared to the US, where the younger population may explain its lower mortality disparity compared to the U.S. pattern [112]. Relatedly, data from Global Health 50/50 revealed that countries with greater gender inequality (e.g., higher gender inequality index, which measures national gender disparities in reproductive health, empowerment, and economic participation ratios) tended to have larger W/M mortality gaps [3]. Conversely, in more gender-equal countries, the difference in COVID-19 mortality between women and men was smaller [14].

A further important factor that complicates a simple narrative of W/M differences is race/ethnicity, and particularly the role of racial disparities in the U.S. context. A short study published as early April 2020, based on data from the U.S. Centers for Disease Control and a survey completed by over 300,000 people, showed that Black, Native American individuals, or those living in low-income households were more likely to have conditions associated with an increased risk of COVID-19 disease, compared to White individuals or those living in higher-income households [113]. Another study of the same cohort showed that residing in neighborhoods with higher proportions of Black residents - a proxy for systematic, concentrated disadvantage in the US context - along with lower educational attainment, poverty, lower median income, and employment disruptions were consistently linked to increased case rates and fatality [114]. Overall, compared to White Americans, COVID-19 mortality rates were nearly four times higher among non-Hispanic Black individuals and three times higher among Hispanic individuals.

While the link between race/ethnicity, socioeconomic conditions, age, and COVID-19 mortality has been highlighted in various reviews and meta-analyses, many such studies did not discuss the intersection with gender or sex [114–116]. Scholars who looked into the variations of racial or socioeconomic disparities by gender have enabled the generation of evidence that sex-related factors can not alone explain W/M disparities. One such study is that of Rushovich et al. (2021), who, motivated by the need to better understand both racial and W/M disparities in COVID-19 mortality, conducted a cross-sectional analysis using state-level U.S. data disaggregated by sex and race [91]. They hypothesized that if W/M differences were primarily biological, W/M disparities would remain consistent across racial groups; conversely, social determinants would produce variation. Their results revealed significant variation in W/M disparities by race, masked in analyses using only one axis of analysis. Black men had the highest mortality rates of all sex/race groups, but notably, Black women also had mortality rates three to four times higher than White or Asian/Pacific Islander men. They further showed that W/M disparities were wider within some racial groups than others, for instance the W/M mortality gap among Black Americans exceeded that among White or Asian/Pacific Islander populations. Moreover, racial disparities within W/M groups were striking: the mortality difference between Black and White women was up to 3.8 times greater than the difference between White men and White women [91]. These findings have been confirmed by other studies [117–120].

Last, many studies on COVID-19 mortality have adjusted for factors such as socioeconomic status, gender, or ethnicity in an effort to explain observed disparities. However, these adjustments are often insufficient. Standard statistical adjustments assume that the effects of gender or ethnicity are uniform across groups, which is rarely the case. Moreover, key explanatory variables such as occupation, housing conditions, or access to healthcare, are frequently missing from health records. This approach can mask crucial intersectional differences, such as how gender disparities manifest differently across ethnic groups or social class, and vice versa, leading to misleading or overly simplified conclusions about the drivers of COVID-19 mortality [108, 121, 122].

## Conclusion

This body of evidence underscores that disparities in COVID-19 mortality between women and men cannot be adequately explained by sex-related factors alone. Instead, they reflect the compounded effects of gendered social roles, occupational exposures, health-related behaviors, and structural inequalities, particularly those related to socioeconomic conditions and race. The intersectionality of these factors amplifies health risks for certain groups, such as socioeconomically disadvantaged men and women from racial and ethnic minorities. Studies that disaggregate data by both gender and race categories reveal significant heterogeneity in outcomes, which is often masked by analyses that adjust for these variables without exploring their interaction. Moreover, international comparisons indicate that gender inequality at the societal level is associated with wider W/M mortality gaps, suggesting that broader social environments, rather than innate biological differences, are key drivers of these disparities. A more specific, intersectional approach allows accurately understanding and addressing sex- and gender-related vulnerabilities in pandemic outcomes.

In challenging the biological sex hypothesis, and considering the role of social environments and inequalities, contextual and structural factors, and their intersection with gender, our purpose is not to assert that sex-related biological factors do not warrant further examination; rather, such factors ought to be considered in their specific context [41], i.e. in interaction with social environments. This approach aligns with recent advances in the study of sex/gender entanglement, which emphasize the need to move beyond artificial separations and to conceptualize biological processes as inherently shaped by social context and environmental factors [1, 41]. Similarly, current evidence suggests that human experiences of COVID-19 likely reflect a complex array of factors, including intersecting disparities and gendered practices and inequities. These factors risk being overlooked when research is guided by a binary, essentialist view of W/M differences.

Importantly, the patterns highlighted here are not unique to COVID-19. Similar dynamics of sex/gender-related disparities, shaped by structural and contextual inequalities, are observed across a wide range of health conditions, including cardiovascular disease, respiratory illness, cancer, and mental health [123]. This underscores the need to integrate a gendered and intersectional approach more broadly in biomedical research, public health, and healthcare policy.

**Table 1:** Biological sex differences in COVID-19 mortality

Category/ Key Elements	Findings/Hypotheses	Evidence & Limitations	Ref.
**Genetic factors**			
X chromosome	Harbors many immune-related genes; women may have an immunological advantage due to mosaic expression and escape from X inactivation	Theoretical advantage for women; lacks robust link to mortality	[44, 46, 70]
Toll-like receptor TLR7	Coded on X chromosome; Protective function	Loss-of-function variants identified; needs further research	[47–49]
Androgen receptor (AR)	Coded on X chromosome; influences antibody production	Suggested link to severe COVID-19 via androgenic alopecia in men; not consistently confirmed	[50, 51, 54]
Genetic polymorphisms	Variants in AR gene (e.g., CAG repeats) linked to severity; genetic polymorphisms	Speculative; later studies did not confirm strong sex-specific associations	[53–57]
Mosaic chromosomal alterations	Loss of Y chromosome associated with increased mortality in men	Linked to immune dysfunction; role still unclear, requires more research	[58, 59] [60]
**Hormonal factors**			
Estrogens vs. Testosterone	Estrogen: immune-activating; Testosterone: immune-suppressing	Animal models suggest estrogen protective; human data inconclusive	[32, 62, 65, 69]
Regulation of ACE2 and TMPRSS2	Estrogen may downregulate ACE2; low androgens may reduce TMPRSS2 expression	Human blood and tissue studies showed inconsistent or no W/M differences; clinical trials inconclusive	[51, 69], 71– [76]
Menopause/Aging	Menopause linked to worse outcomes; aging men with low testosterone more vulnerable	Observational data; led to trials (estradiol, progesterone, anti-androgens), many with mixed or halted results	[53, 63, 64], 66– [69]
**Immunological factors**			
X-linked immune genes	Contribute to stronger immune responses in women	May lead to better pathogen clearance, stronger antiviral responses	[5, 46, 77]
Inflammatory response	Men show more hyperinflammation; women show better T-cell responses	Mixed evidence; not consistently correlated with mortality differences	[35–37, 43, 53]
Full immune response cascade	Studies examined sex differences in IFN response, cytokines, T cells, antibodies	Results inconsistent and sometimes contradictory; mechanisms speculative	[43, 47] [61, 69], 79– [81]

## Data Availability

No datasets were generated or analysed during the current study.

## Abbreviations

ACE2: Angiotensin-Converting Enzyme 2
BMI: Body Mass Index
COVID-19: Coronavirus Disease 2019
GII: Gender Inequality Index
ICU: Intensive Care Unit
M/W: Men/Women
SARS-CoV-2: Severe Acute Respiratory Syndrome Coronavirus 2
TMPRSS2: Transmembrane Protease Serine 2
W/M: Women/Men

## Footnotes

1 We recognize that researchers regularly usefemale/male to classify research participants, in both human and non-humanpopulations. In this review, we use women/men (W/M) when referring to humanstudies, to reflect the intersection of gender/sex in human health experiences.
